# Crossmodal influences on food perception in those with smell and taste disorders: A preliminary study

**DOI:** 10.1371/journal.pone.0358651

**Published:** 2026-10-01

**Authors:** Lorenzo D. Stafford, Abigail Morris, Duncan Boak, Carl Philpott

**Affiliations:** 1 Department of Psychology, University of Portsmouth, United Kingdom; 2 SmellTaste, United Kingdom; 3 UEA, Norwich, England; Universidad Nacional de Tres de Febrero, ARGENTINA

## Abstract

**Objectives:**

Individuals living with Smell and Taste Disorders (SATDs) have a reduced quality of life in a number of different ways with one central factor being their inability to appreciate food flavour and thereby have a less fulfilling eating experience. Separately, it has been demonstrated that the perception of food can be influenced by senses beyond smell and taste. The aim of the current study was therefore to explore whether altering the way in which food is presented, influences its perception for those with SATDs.

**Methods:**

Participants (n = 33) who self-identified as living with smell loss, completed a standardised olfactory threshold test, followed by a food taste test where different foods were consumed in three different modalities (Vision-colour, Shape-food shape, Sound-music mood) and ratings made on hedonic and sensory dimensions. We also measured the impact of smell loss using the Olfactory Disorders Questionnaire (ODQ).

**Results:**

Within each modality, we found no clear evidence for contrasts in hedonic and sensory ratings, when food was presented differently. In contrast, the qualitative findings revealed that since losing their sense of smell, the most common practice for individuals to make eating food more enjoyable was the use of different food texture and additional herbs/spices.

**Conclusion:**

As far as we are aware, this is the first study to examine whether food experience for those with SATDs can be influenced by using different modalities. The findings revealed a difference between the quantitative and qualitative data which suggests that more carefully designed studies are needed to detect effects.

## Introduction

It is estimated that around 5 percent of people in a given population have no sense of smell [1], which carries an increased risk from a range of hazards including the inability to detect gas leaks and foods that are unsafe to consume [2]. To many affected by smell and taste disorders (SATDs), being unable to smell is a disability with significant changes to their quality of life (QoL) [3,4], with awareness of this condition increasing dramatically since the global Covid-19 pandemic. One key aspect, frequently reported by those with SATDs is the loss of enjoyment of food [5], which can result in weight disturbances with roughly equal numbers losing/gaining weight [5]. It appears that for some, not being able to experience the flavour (interaction of olfactory, gustatory and trigeminal function) of food leads to reduced interest in food and appetite, whereas for others, their impairment leads them to consume more food, possibly, as a means of compensating for the reduced pleasure in flavour perception [3,6]. The importance of flavour is further demonstrated by work where individuals with SATDs completed objective tests measuring orthonasal (odours outside of the mouth) and retronasal (odours and thus flavour inside the mouth) function [7]. Key findings were that whilst increases in performance in both tests were associated with a better QoL and lower depression, the latter accounted for more of the variance. Since measures of QoL include many items relating to food, this strongly suggest the link between flavour function and QoL. This then raises the question of whether there is any way of improving eating experiences for those with SATDs.

The idea that the perception of food could be influenced by other senses (especially sound) to any significant degree might have appeared as unlikely at one time; however, a now classic experiment demonstrated how the taste and appreciation of potato crisps can be manipulated by altering the crisp masticating sound volume [8]. In addition to altering the food sound itself, work has also shown how environmental sound (especially music) can influence the perception of food (e.g., [9]) and most recently, music’s ability to increase perceived sweetness in foods and thereby encourage healthier eating (i.e., of less sweet but healthy foods) [10]. In the intervening years, a number of studies have extended the domain to show how colour can also yield similar effects, where for instance food served in different coloured bowls led to contrasts dependent on colour and hedonic/sensory dimension [11]. It was found that popcorn was rated as sweeter when served in a red bowl, but saltier in a blue coloured bowl. It has been theorized that this is due to the particular colour flavour association where colours such as blue and red are associated with basic tastes of salt and sweet respectively [12,13]. In terms of texture, work has also shown how manipulating both the mouthfeel (intrinsic) and handfeel (extrinsic) aspects can influence various dimensions of food [14].

To our knowledge, no research has examined whether such effects also emerge in those with SATDs; thus, it remains unknown whether food enjoyment is altered by manipulating the sensory characteristics of the eating experience. We theorise that the possible mechanism for any such effect is based at least in part, on the previously learned association between the food and the multisensory component(s). Hence prior to onset of the SATD, individuals would be exposed to the same sorts of food associations as anyone else and thereby learned that for instance, blue is associated to salty foods [12]. However, what remains unclear is the longevity of such learned associations and whether they can induce changes to food perception in absence of the ability to detect the complete flavour. Drawing on broader research from other disabilities, there is evidence that in blind individuals, stimulation in other senses such as audition and touch, results in neural activity in visual parts of the brain (see reviews [15,16]), but as this also seen in congenital blindness, it suggests those crossmodal effects are not based on learned associations. It is also important to note that whilst heightened sensitivity (sensory compensation) has been found in other disabilities (e.g., blindness, see review [16]), this does not seem to be the case for SATDs [17]; hence, it is not the case that those with SATD consistently develop heightened perception in their other senses. Logically then, any resultant crossmodal (Multisensory) effects in SATDs are likely due to individual changes in flavour perception, rather than any more generic alterations.

We therefore aimed to explore this through an experiment where we recruited individuals with SATDs and first tested their odour function using a standardized odour threshold test [18,19]. They were then asked to rate different foods for hedonic and sensory properties in contrasting modalities (Vision/Shape/Sound). Due to the absence of any previous research, no firm predictions could be made and so the main aim was to explore any such differences.

## Method

### Participants

Participants (n = 33; *M*_age_ = 63.5, *SD*_age_ = 12.8; range 19−85; 19 females/14 males) were recruited through an advert on the University website and separately via the charity ‘SmellTaste’(A UK charity that supports people who experience smell and taste disorders; Formerly known as FifthSense). The study was described as examining the perception of food in those individuals living with no (or severely impaired) sense of smell. The inclusion criteria required individuals to be in good physical health and due to the nature of the study, not have respiratory problems (e.g., Asthma) or food (including nut) allergies or food intolerance (e.g., gluten) and not suffer from colour vision deficiency. Participants were recruited between 1/7/2023 and 18/8/2023. The study received ethical approval from the University’s Science Faculty Ethics committee (SHFEC 2023−044) and written informed consent was obtained from all participants. Participants were paid £15 for participating in the study.

### Design

This study utilised a within-subjects design to examine the effects of modality on food perception. Participants completed 3 different food tasting tests where for each test, the same food was presented in three different conditions (Types); Vision (Colour of plate): Blue, Green, Yellow; Shape (Shape of food): circle, triangle, clover; Sound (Mood of music accompaniement): Neutral, Sad, Fear. Hence for Vision and Sound, we manipulated ‘extrinsic’ cues, whereas for Shape, we altered the ‘intrinsic’ cues. We used a latin-square to counterbalance the test order for Modality (Vision/Shape/Sound) and for food presentation. The main dependent variables were the sensory and hedonic ratings of the food. Sample size was estimated by a power analysis (G*Power 3.1 [20],), which for RM ANOVA, given a medium effect size, power 0.8 and α = 0.05, recommends N = 30 participants. To account for possible attrition, we aimed to recruit in excess of 30 participants.

### Materials

#### Olfactory Threshold Test.

For the threshold test, the odour, n-butanol (Fisher Scientific, UK), was diluted in distilled water using a two-fold serial dilution in 16 steps. Using fifteen 50 ml amber glass bottles, 1% concentration (step 1) was diluted by a factor of two for subsequent steps to form the lowest concentration (step 16). Two ‘blank’ bottles containing dilutant alone were incorporated into each dilution step alongside the odorant to create triplets for the purposes of the test. To begin, the bottle with the highest concentration (target odour) was presented to participants to enable its recognition throughout the test. Like all the bottles in this test, the target odour was waved approximately 2 cm below the nostrils for 3–4 seconds to allow for a natural but optimal inhalation. Throughout, the participants were exposed to multiple sets of three counterbalanced bottles. Due to participants wearing a blind fold, the researcher would say “odour one” etc. when the bottle was alternating below each nostril to increase awareness of its location. Following presentation of the last bottle of the triplet (counterbalanced), participants were asked which bottle contained the odour (1, 2 or 3). If the participant answered correctly (and it was the lowest concentration), they were presented with the same triplet again (in a different order) and the task repeated until they made a mistake, which resulted in the triplet containing the next concentration step being presented. Using a single up-down staircase system (as used widely in olfactory research, e.g., [18]), this was then repeated until there were seven ‘turning points’, with the mean of the last four points determining the threshold score (TS) for the individual. Throughout, the participant wore a blindfold to prevent identification of the odours and the researcher wore cotton gloves (Boots, Portsmouth) to avoid odour cross-contamination.

### Vision

We completed a pilot study where 12 participants (2 male and 10 female, who took no part in main study) were asked to rate biscuits (Tesco’s Oaty Rounds Biscuits;15g, 70kcal per biscuit) which were broken into quarters and two placed into six different coloured bowls (Pink, Green, Yellow, Orange, Blue, Light Green) (Asda supermarket). Ratings were made using VAS (Visual Analogue Scales) for pleasantness and sensory dimensions (flavour intensity, sweet, bitter, sour, salty). On the basis of those findings, we chose the three coloured bowls (green, blue, yellow), with the largest differences in primarily pleasantness/saltiness, for the main study.

### Shape

As part of the same pilot study, participants completed ratings for specific shaped (circles, triangles, clover, star, heart, hexagon) snacks (Asda Cheese Savouries Sharing Snacks; per 1/10 pack: 25g, 129kcal), where two of each shape were placed into six different coloured bowls.

To disguise the fact that the snacks were all essentially the same savoury food (differing only by shape), participants wore a blindfold during the tasting of the snack which was then removed to complete the ratings. The three shapes creating the largest differences in VAS ratings were chosen for the main study (circle, triangle, clover).

### Sound/Music

In a second pilot study, 7 participants (3 male and 4 female^2^) completed ratings for food items (Tesco’s Pineapple Chunks in Juice; per ½ can: 212g, 108kcal), where one chunk was placed into four different coloured bowls. Participants wore headphones (Oneodio) and whilst tasting each of the three foods, listened to a different type (mood) of music (YouTube) via a laptop (Acer TravelMate). We used classical music since there is extensive research showing its reliability to induce different mood states (e.g., [21]). Neutral mood music comprised of “The Planets, Op. 32: VII. Neptune – The Mystic” (Holst) (Royal Liverpool Philharmonic Orchestra – Topic, 2017) per previous work [22]. For happy music, we used A Musical Snuff-Box, Op. 32” (Lyadov) (Vladimir Ashkenazy – Topic, 2018); Sad music using “Waltz No. 9 in A-Flat Major, Op. Posth. 69 No. 1 “Farewell”” (Chopin) (Cyprien Katsaris – Topic, 2017); Fear music Mephisto – Waltz No.1” (Liszt) (André Laplante – Topic, 2015) to induce a fearful mood [23]. As in the shape condition, to disguise the fact that the snacks were all the same food, participants wore a blindfold during the tasting which was then removed to complete the ratings. We selected the three types of music yielding the largest differences in VAS ratings (Neutral, Sad, Fear) for the main study.

For the main study, participants were presented with three different types of food (biscuits, savoury flavoured snacks, pineapple chunks) in three different conditions that manipulated modality (vision-bowl colour, shape-food shape, sound-music). Hence, each participant sampled nine food items (i.e., 3 x biscuits, 3 x savoury snacks, 3 x pineapple chunks). The pairing of food to modality (e.g., biscuits-vision) was consistent across participants.

### Food Ratings

VAS forms were provided to participants after each bowl of food. Each form displayed six descriptors (pleasant, salty, sweet, flavour intensity, sour, bitter) and their corresponding scales 100 millimetres in length. Participants had to draw a vertical line on each scale between “Not at all” and “Extremely” to indicate their opinion of the food item in relation to that descriptor. Each marked scale could be measured in millimetres, with 0 mm (“Not at all”) indicating a low perception of the food descriptor, and 100 mm (“Extremely”) indicating high levels.

### Olfactory Disorders Questionnaire (ODQ)

The ODQ adapted for English speakers [24] was used in this study to measure quality of life impairments in those with olfactory dysfunction. Participants were asked to tick whether they “Agree”, “Agree Partly”, “Disagree Partly”, or “Disagree” with each of the 24 statements presented. These answers were scored between 4−0 respectively. Examples of the statements includes “I am scared of getting exposed to certain dangers (e.g., gas, rotten food)” and “I go to restaurants less often than I used to”. For question 9 which stated “I have gained (G) or lost (L) weight”, participants were advised to write ‘G’ OR ‘L’ and if they thought their weight had not changed, then write ‘Not Changed’. As with the original scoring of the scale [25], overall high scores indicated lower quality of life, as a result of smell loss.

### Food eating practices questionnaire (FEPQ)

To gain insight into the food eating practices of those with olfactory dysfunction, the researchers developed the FEPQ to use within the study. This required written responses and vertical lines on horizontal 100 mm VAS scales to rate whether each statement was relevant to them “Not at all” (0) or “Extremely” (100). Examples of some questions that were presented to participants include “Since you have lost your sense of smell, what strategies do you commonly use to make eating food more enjoyable? (Tick all that apply)” followed by statements to tick and corresponding boxes to allow for written explanations, e.g., “Use Different Food Textures, e.g., crunchy salads. If so, which?”. Participants also used separate VAS scales to rate how important different aspects of food practices were for them, e.g., “Cooking/Food Preparation”. Finally, they were asked “In your own words, please describe anything else about your current eating practices that you think could be useful for us (or for others with anosmia) to know” to allow the researchers to gain insight into other unique practices that may be useful to consider further.

### Procedure

Participants were advised to avoid smoking, eating, or drinking anything other than water at least two hours prior to the study. Upon arrival, they completed the ODQ, followed by the threshold test. Next, they completed the Food tests and were advised that a bowl containing a food type would be placed in front of them, which they would then taste and complete the rating (VAS) form provided. They were advised that it was not a requirement to finish all of the snack in the bowl and were instructed to sip some of the water provided to them in between bowl presentations. For the Shape modality condition, participants were required to wear a blindfold and asked to place their hands on the desk to enable the researcher to gently touch the participants hand with the bowl of food to increase awareness of its location. They were then able to pick up the shape and place in their mouth. Upon request, participants waited for the researcher to remove the bowl before removing the blindfold to rate the bowl of food. After sipping the water, they were asked to replace their blindfold and repeated the steps for the remaining food/modality. For the Sound modality condition, the participant wore headphones (and a blindfold) and the researcher completed a test to ensure the sound was audible. It was then described that the researcher would play a sound for 45 seconds and during this period, they were instructed to taste the food and complete the ratings. Once the three modality food tests had finished, participants completed the FEPQ followed by a full debriefing. The study took approximately 60 minutes to complete.

### Data Analyses

For the ODQ(Q9), we observed that for the vast majority of participants, weight had not changed and we therefore analysed this question separately from the overall ODQ score.

Looking at the sample overall and age of olfactory dysfunction, it was reasoned that since individuals with congenital smell loss (n = 3) had never had the opportunity to learn an association between modality and flavour, their food rating data would be different to those with acquired SATDs and were therefore excluded from the food rating analyses. Preliminary screening of the food rating data revealed a consistent multivariate outlier whose data were also removed.

For the food tests, the hedonic and sensory dimensions were analysed for each modality (vision, shape, sound,) using a Repeated Measures ANCOVA, with the within-subjects factor of Type (3 levels) and Threshold score (continuous variable) as a covariate. We defined families of hypotheses, whereby: 1. Main effect of Type; 2. Main effect of Threshold; 3. Type x Threshold interaction and applied a Holm correction to each omnibus ANCOVA (see Supplementary Materials for uncorrected values/analyses). Follow up tests were adjusted for multiple comparisons (Sidak).

## Results

### Sample Characteristics

The sample comprised of primarily middle-aged individuals which is typical of the research in this domain with the general trend for smell loss to increase with age (Table 1). The level of impairment as a result of olfactory dysfunction (total ODQ score) was lower in the sample here (M = 24.6) when compared to a previous UK sample using hospital outpatients, prior to treatment (M = 54.2, comprises all 24 questions, including Q9 [24].). In an effort to further explore the relation between quality of life and variations in odour function, we analysed the ODQ data in terms of congenital, acquired anosmia and respective Threshold Score (TS = 0, TS > 0), resulting in three groups: Congenital (N = 3, all TS = 0), Acquired (N = 9, TS = 0), Acquired (N = 20, TS > 0). Analysis revealed a main effect of Group, F(1, 29)=4.44, p = .02, η² = .23, where mean comparisons revealed higher ODQ scores (lower quality of life) in the Acquired (TS > 0) group versus the Congenital group, (Fig 1). The pattern of Acquired anosmics having a lower quality of life compared to Congenitals has also been found in previous work (e.g., [26]).

**Table 1:** Mean (SD) participant characteristics (n = 33).

Measure	Frequency/Mean (whole sample, n = 33)		Frequency/Mean (final sample, n = 29)
Age	63.5 (12.8)		64.6 (9.9)
Gender (F/M)	19/14		17/12
ODQ (exc Q9-weight gain)	24.6 (10.8)		25.8 (10.3)
ODQ-Q9 (weight Change)			
No Change	23		19
Gained weight	5		5
Lost weight	3		3
Threshold Score	4.2 (3.9)		4.7 (3.9)
Cause Of Odour Dysfunction:			
Unknown	18		14
COVID-19	6		6
Head Injury	3		3
Diagnosed Idiopathic	2		2
Stress	1		1
Age	1		1
Chemical Exposure	1		1
Post Operative	1		1
Type Of Odour Dysfunction:			
Undiagnosed	20		17
Not Specified	4		4
Anosmia	3		2
Hyposmia	3		3
Phantosmia-Hyposmia	2		2
Phantosmia	1		1
Age Onset Of Odour Dysfunction:		**ODQ (exc Q9)**	
Congenital	3	10 (6.6)	
< 30 yrs	3	28 (17.5)	
31-40 41-50	5 4	27.6 (7.4) 16.5 (5.1)	
51-60	9	30.3 (12.0)	
61-70	4	26.5 (6.8)	
71-80	2	30.5 (4.9)	
Unknown	3	19.7 (6.5)	

Notes

ODQ-Q9: Two responses were ambiguous and were therefore unable to be categorised

Threshold Score: These values can be compared to an undergraduate student sample who completed the same olfactory test (M = 10.0 ± 0.26).

**Fig 1:**
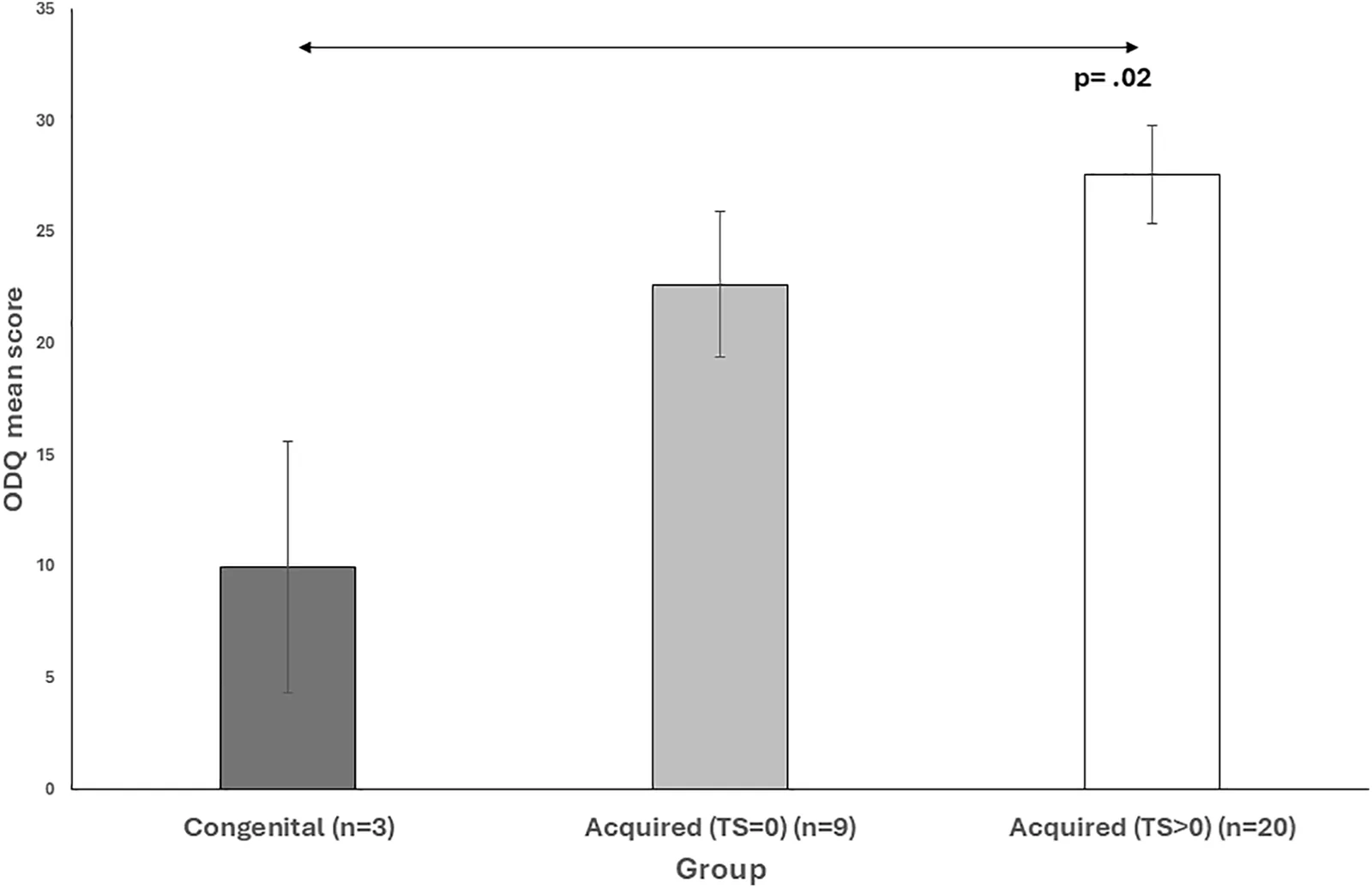
Mean (SE) ODQ Scores By Group (TS = Threshold Score).

### Modality and pleasantness

#### Vision.

The main effect of Colour Type was not significant, F(2, 54)=1.15, p = .95). The main effect of Threshold score (covariate) and interaction with Colour Type were also not significant [F < 1, p = .98; F < 1, p = 1.00] (Table 2).

**Table 2:** Mean (SD) food hedonic and sensory ratings with threshold score as covariate (n = 29).

	Pleasantness	Salty	Sweet	Flavour Intensity	Sour	Bitter
**Modality**						
**Vision-Colour**						
Green	59.3 (24.9)	9.1 (12.7)	54.0 (27.0)	31.6 (25.9)	2.2 (2.1)	2.6 (2.6)
Blue	61.4 (24.0)	6.9 (10.1)	56.9 (25.3)	37.9 (28.4)	2.4 (2.6)	2.6 (2.9)
Yellow	57.9 (24.9)	11.0 (16.1)	50.3 (27.6)	34.6 (27.8)	2.4 (2.6)	3.1 (4.3)
**Food Shape**						
Circle	50.1 (23.2)	36.1 (27.7)	8.0 (11.6)	24.2 (22.6)	7.6 (12.4)	5.7 (10.0)
Triangle	51.3 (17.4)	41.6 (32.5)	12.1 (15.2)	29.5 (23.2)	4.9 (7.3)	4.7 (6.1)
Clover	45.6 (24.5)	40.6 (26.0)	7.8 (10.4)	27.5 (24.2)	5.1 (9.9)	5.2 (7.6)
**Sound-Music**						
Neutral	62.2 (22.4)	2.7 (2.9)	51.8 (28.5)	38.4 (31.6)	16.7 (24.6)	10.3 (21.2)
Sad	53.2 (21.9)	3.1 (4.9)	48.4 (23.3)	35.6 (27.6)	15.0 (22.9)	10.4 (19.9)
Fear	58.7 (23.5)	2.6 (2.5)	53.3 (29.2)	41.8 (29.2)	19.3 (25.3)	13.0 (24.0)

**Notes**

Vision-Colour: Due missing data for one participant (salty/sweet), analyses for these data were restricted to 28 participants

Sound-Music: Due missing data for one participant (neutral music), analyses for these data were restricted to 28 participants

#### Shape.

No significant main effects were found for Shape Type, F(2, 52)=1.44, p = 1.00) or Threshold score (covariate), F < 1, p = 1.00). The interaction of Shape Type and Threshold score was also not significant, F < 1, p = 1.00.

#### Sound/Music.

The main effect of Sound Type and its interaction with Threshold score (covariate) were not significant F(2, 52)=0.98, p = 1.00; F(2, 52)=0.003, p = 1.00). The main effect of Threshold score approached significance, F(1, 26)=7.65, p = .06.

### Modality and sensory dimensions

#### Vision.

For saltiness, the main effect of Colour Type was not significant, F(2, 52)=4.14, p = .13 and the main effect of Threshold score F(1, 26)=1.29, p = .80, and Colour Type x Threshold interaction, were also not significant, F(2, 52)=3.35, p = .26.

The corrected or uncorrected analyses for Sweet, Flavour, Sour and Bitter were not significant.

#### Shape.

No significant corrected or uncorrected effects were found.

#### Sound.

For sweetness, the main effects of Sound Type and Threshold were not significant [F < 1, p = 1.00; F(1, 26)=6.29, p = .09], and neither was the interaction [F = 1.25, p = 1.00].

The corrected or uncorrected analyses for Saltiness, Flavour, Sour and Bitter were not significant.

### Food eating practices

The data from the FEPQ is summarized in Table 3 and we observed that the most common strategies participants use to make food more enjoyable are centred on texture and the use of herbs/spices. The final question on the 3 aspects of food data was interesting in the rather modest but variable (Ms < 58) ratings which could be linked to smell loss.

**Table 3:** Frequency Of responses to food eating practices questionnaire (FEPQ) (n = 33).

	Total Frequency	Examples (most popular)
**Q: Since you have lost your sense of smell, what strategies do you commonly use to make eating food more enjoyable?**		
Use Different Food Textures	18	Crunchy, Fruits, Salads
Add ‘extra’ herbs/spices	20	Cumin, Paprika, Chilli flakes, Chilli
Visual presentation of food,	6	Colourful visuals, avoids beige dinners
Play music whilst eating food	7	Relaxing, Rock, Pop
Watch programmes/films whilst eating food	5	Adventure, Action, Science-fiction
Any other practices	17	Preference for highly salty foods, Preference for very sweet foods
**Q: Which one do you use most frequently**		
Strong emphasis on the importance of texture	2	
Eat because they need to, not because they want to	2	
**Q:How important are the following aspects of food for you:**	**Mean (SD)**	
Cooking/Food Preparation	57.5(SD = 32.9)	
Food Flavour	54.4 (SD = 37.0)	
Overall Eating Experience	54.9 (SD = 31.7)	

## Discussion

In this study, we examined the influence of modality on food perception in those with SATDs and after applying statistical correction for multiple tests, found no clear evidence for any effects. This was particularly surprising in the visual modality, given the findings from previous research where for instance, sweet popcorn served from a blue versus white bowl was rated as saltier, whereas salty popcorn was rated sweeter, served from a red compared to white bowl [11]. However, work has also shown mixed evidence for plateware colour to influence food perception; for example, one study found colour-food effects were restricted to those categorised as Picky Eaters [27].

In terms of the sound modality, we did not find any differences between the three music conditions, which is in contrast to broader research, where for instance, individuals rated beer as less pleasant in a negative versus positive music emotion condition [28]. In trying to account for these differences, it could be that in the work here, the inclusion of two mood conditions (Sad/Fear) of a similar negative affect did not provide sufficient sensitivity to detect any effects. The decision to use fear inducing music was led by our initial pilot work; however, upon reflection, utilising a more contrasting ‘positive’ mood music, may have yielded clearer differences, as found in the broader literature [29].

In terms of shape, we did not find any influence on the perception on either hedonics or sensory dimensions of food. In contrast, for those without SATDs, there is a wide body of work demonstrating how both intrinsic and extrinsic manipulations of texture can affect perception [14,30]. For instance, one study found that increasing the hardness of food mouthfeel led to reductions in flavour intensity [31]. It is therefore possible that using the same logic, that softer foods might increase flavour intensity for those with SATDs. Linked to these findings, it was interesting that in the FEPQ, texture was stated as one of the most consistently used ways of making food more enjoyable (e.g., salads), whereas in the study here, no effects were observed in food pleasantness of different shape textures. In trying to reconcile this finding, it could simply be that the type of food used in the present study did not offer sufficient sensitivity to detect such differences, as say compared to crunchy versus softer salads, where there is some evidence to suggest differences might be observed [32]. It is also important to note in the present study, that whereas for vision and sound where we manipulated extrinsically (plateware colour, music accompaniment), in contrast for shape, we used intrinsic cues in terms of the different shape geometry of the food itself. A further possibility is that the findings here represent the known differences between individuals with SATDs. Relevant here, two recent studies yielded contrasting findings, with one showing that those with olfactory impairment rated food taste and texture as less important than controls [33], whilst another study found an increased awareness of taste and mouthfeel following smell loss [34]. As has been suggested (e.g., [33]), it could be that for some individuals, a reduced focus on taste/texture could be part of a coping mechanism that helps some individuals come to terms with their disability, whereas for others with perhaps a more intact sense of smell, the increased focus enables them to maximise their food enjoyment.

The FEPQ findings further showed that the most frequently reported strategy to make food more enjoyable was to add extra herbs/spices, which agrees with previous research that found 39% of those with Olfactory loss increased their use of spices, since losing their sense of smell [35], which has also been linked to being less sensitive to the chemesthesis reaction produced by such spices [34]. By asking participants to provide examples, the current study also revealed the most common specific spices used were ‘Cumin, Paprika, Chilli flakes, Chilli’. The inclusion of chilli (capsaicin) would be expected, due to its ability to induce the characteristic pungent burning/tingling sensation via trigeminal nerve activation [36], that is an important dimension of food flavour and enjoyment for many people. However, the presence of the other spices (cumin, paprika) that do not produce the same intense sensation is interesting, though work has referenced similar sensations (burning/tingling) in cumin [36]. One could also theorise the inclusion of these spices might also be part of a more general association with particular foods that are co-used habitually, e.g., it is common for South-Asian curry recipes that require chilli to also need cumin. Hence, it could be that for some anosmics, the addition of some spices/herbs, even if not yielding an objective olfactory or chemisthetic response, nevertheless increase food flavour perception. Providing some support for this, one recent study has examined the ability of spices to enhance the sensory/hedonic dimensions of food for those with SATDs [37]. In that work, the same food was perceived as saltier, more intense and more liked, when it contained a moderate level versus no capsaicin. The rationale for that study was using spice-associations to reduce the excess use of salt but also serves to demonstrate a further example of multisensory effects on food perception, when one is reminded that flavour engages many different systems (olfactory, gustatory, trigeminal).

In terms of the study limitations, since only one type of food was used for each modality, we cannot be sure whether different foods might yield different effects. The main rationale for this was due to practicalities of keeping the food constant but only manipulating the modality type (e.g., bowl colour); hence testing another food with colour would have required another 3 presentations and the same for Shape and Sound which could lead to possible fatigue/demand characteristics. More broadly speaking, we recognise that the choice of foods used in the study may not be that representative of the foods generally consumed by those with SATDs. Hence a more ecologically valid food for testing (for instance) shape, would be to look at salads of differing texture, especially as a number of individuals already use this strategy to increase food enjoyment (see Table 3). However, whilst future work is encouraged to include such foods, a balance will need to be accepted between ecological validity and the constraints of the experimental method. It is also important to note that the visual (modality) did not include a control (i.e., white bowl colour) condition. The rationale here was that our study was not examining whether coloured bowls differ from a neutral baseline, since that has already been shown in previous work (e.g., [11]; see also a review [38],), but instead we explored differences in coloured bowl conditions which were informed by our extensive pre-study pilot. Though we do see the value of retaining a control and recommend that be considered for future work.

In terms of the study sample size, though this was appropriate per the power analysis prior to the study, we acknowledge that given the number of analyses and need for multiplicity corrections, a larger sample would have provided greater sensitivity to detect any effects. Separately, since we used a newly created and therefore unstandardised measure for Food Eating Practices (FEPQ), those findings should be considered as preliminary. Finally, though we completed a test of participants ‘olfactory’ function, it would also be informative, given the relation to food and flavour, to complete a test of gustatory (taste) function and separately, a test of retronasal function, such as the Candy Smell Test [39] or Flavour Test [40], and on a related note, it would have been prudent to assess participants hearing ability, as this may have affected performance in the music modality.

In what we believe is the first study to examine this phenomenon, we found no clear evidence for cross-modal influences on food perception in those with SATDs. However, participants did report that habitual use of texture as one of the main strategies to make food more enjoyable, which suggests that modality is indeed important. Since eating behaviour is one of the key factors affected by smell and taste loss, which can lead to weight loss (but also weight gain) [5], we encourage more research in this area to understand further how our senses beyond smell and taste, can be used to enhance food eating experiences.

## Supporting information

S1 Text Analyses Without Holm Adjustment. (DOCX)
