# Supplementary material for: Crossmodal influences on food perception in those with smell and taste disorders: A preliminary study
Source: PLoS One. 2026 Oct 1;21(10):e0358651. doi: 10.1371/journal.pone.0358651 (PMC13630209; doi:10.1371/journal.pone.0358651)
Supplement: Supplementary file 1 [file pone.0358651.s001.docx]

**Supplementary Materials**

Analyses Without Holm Adjustment:

*Modality and pleasantness*

*Vision*

The main effect of Colour Type was not significant, F(2, 54)=1.15, p=.32), The main effect of Threshold score (covariate) and interaction with Colour Type were not significant [F<1, p=.98; F<1, p=.66].

*Shape*

No significant main effects were found for Shape Type, F(2, 52)=1.44, p=.25) or Threshold score (covariate), F<1, p=.48). The interaction of Shape Type and Threshold score was not significant, F<1, p=.58.

*Sound/Music*

The main effect of Sound Type and its interaction with Threshold score (covariate) were not significant F(2, 52)=0.98, p=.38; F(2, 52)=0.003, p=.99), though we did find a main effect of Threshold score, F(1, 26)=7.65, p=.01, η² = .23.

*Modality and sensory dimensions*

*Vision*

For saltiness, the main effect of Colour Type was significant, F(2, 52)=4.14, p=.02, η² = .14, with mean comparisons revealing a non-significant trend toward higher ratings for the yellow versus blue (p=.15, NS) condition. The main effect of Threshold score was not significant F(1, 26)=1.29, p=.27, but we did find a significant Colour Type x Threshold interaction, F(2, 52)=3.35, p=.04, η² = .11. The analyses for Sweet, Flavour, Sour and Bitter were not significant.

*Shape*

No significant effects found.

*Sound*

For sweetness, the main effect of Sound Type and its interaction with Threshold score (covariate) were not significant [F<1, p=.87; F=1.25, p=.30]; but there was a main effect of Threshold score, F(1, 26)=6.29, p=.02, η² = .20.
